# Harnessing digital health interventions to address the heterogeneity of depression: a systematic review

**DOI:** 10.3389/fdgth.2025.1654745

**Published:** 2025-11-18

**Authors:** Ghufran Alsalloum, Sarah Dalibalta, Yacine Hadijat

**Affiliations:** 1 Department of Bioscience and Bioengineering, College of Engineering, American University of Sharjah, Sharjah, United Arab Emirates; 2 Department of Biology, Chemistry and Environmental Sciences, College of Arts and Sciences, American University of Sharjah, Sharjah, United Arab Emirates; 3 Innovation in Health Science and Digital Health, College of Medicine, Mohammed Bin Rashid University of Medicine and Health Sciences, Dubai, United Arab Emirates

**Keywords:** depression, digital mental health, personalization, heterogeneity, precision psychiatry, systematic review

## Abstract

**Background:**

Depression affects over 229 million people worldwide and ranks among the leading causes of disability and death, particularly in young adults, where suicide is a top contributor to mortality. Standard diagnostic and treatment approaches often overlook the marked clinical and biological heterogeneity of depression, resulting in low first-line remission rates and prolonged trial-and-error care, underscoring an urgent need for precision strategies in mental health practice.

**Objective:**

This review explores the recent literature (January 2020–September 2025) on personalized digital health interventions for depression, with an emphasis on how these technologies address heterogeneity in symptomatology, biological underpinnings, and treatment response across diverse patient populations.

**Methods:**

The study followed PRISMA guidelines, searching Scopus, IEEE Xplore, and ClinicalTrials.gov for English-language peer-reviewed articles and trials published and registered between January 2020 and September 2025. Only studies relevant to depression heterogeneity and digital health were included, and studies focusing solely on generic digital health tools without a personalized or adaptive component were excluded. Findings were synthesized narratively.

**Findings:**

29 publications were reviewed: 20 studies and 9 clinical trial reports, representing over 5,000 participants. Personalized machine-learning models using mobile sensing and ecological momentary assessments improved mood-forecasting accuracy by up to 25%. Randomized trials of just-in-time adaptive interventions (e.g., the Mello app) demonstrated moderate to large effect sizes for reductions in depression (*d* = 0.50), anxiety (*d* = 0.61), and repetitive negative thinking (RNT) (*d* = 0.87). Smart-messaging post-Cognitive Behavioral Therapy yielded sustained well-being improvements over 12 months, while neuromodulation-based digital therapeutics targeting apathy networks in late-life depression showed significant gains in executive function and motivation. Most studies featured small, convenience samples, variable outcome measures, and limited external validation; risk-of-bias concerns included lack of blinding and incomplete handling of missing data. Equity analyses across demographic and clinical subgroups were seldom reported.

**Conclusions:**

and Relevance: Digital mental health technologies exhibit substantial promise for delivering personalized interventions that accommodate inter-individual variability in depression. High-quality evidence supports their capacity to enhance prediction, engagement, and clinical outcomes. However, broader implementation requires standardized multidimensional outcome measures, equity-focused algorithm validation, and integration of established clinical phenotypes.

## 1 Introduction

Depression affects approximately 229 million people globally, imposing a heavy toll on individuals and society (1). In 2021, suicide, often linked to severe depression, claimed over 700,000 lives and ranked as the third leading cause of death among individuals aged 15–29 (2, 3). The disorder significantly diminishes productivity in work, education, and relationships, with depression and anxiety together accounting for 12 billion lost workdays annually and nearly $1 trillion in global economic costs (4). The Coronavirus disease (COVID-19) pandemic further intensified this burden, with depressive disorders rising by 18% and anxiety disorders by 15% from 2019 to 2020 (5). By 2030, depressive disorders are projected to become the 12th leading cause of death, with a 35% increase in Disability-Adjusted Life Years (DALYs) (6).

### 1.1 Heterogeneity of depression

**Figure 1:**
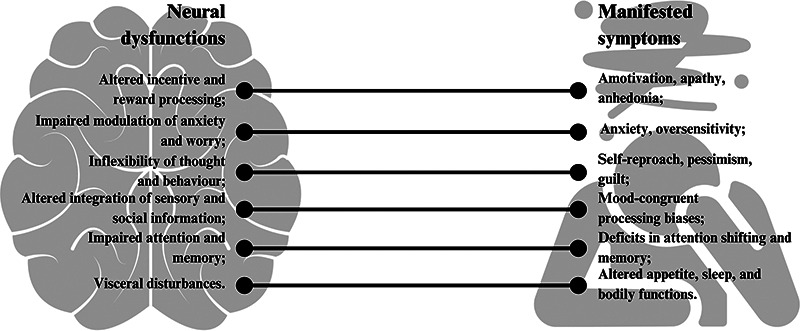
Mapping of neural dysfunction with manifestations of depressive episodes.

Depression manifests with remarkable variability across individuals, presenting diverse symptom profiles, disease trajectories, and treatment responses that complicate standardized approaches (7). This heterogeneity extends to biological foundations, as research demonstrates that genetic polymorphisms, hormonal factors, inflammatory markers, and neural connectivity patterns all contribute to individual differences in depression presentation (8). Key symptoms of depressive disorders include reduced motivation and pleasure (anhedonia), difficulties in managing anxiety and worry, inflexible thought patterns leading to self-reproach and guilt, impaired processing of sensory and social information, cognitive deficits in attention and memory, and various physical disturbances such as changes in weight, appetite, and sleep patterns (9). Figure 1 maps the different neural dysfunctions associated with depressive episodes and their manifested symptoms (9, 10).

Genetic evidence strongly supports the notion that depression comprises multiple biologically distinct subtypes. For instance, early-onset, recurrent, and postpartum depression exhibit higher Single Nucleotide Polymorphisms (SNP)-based heritability, indicating stronger genetic loading. Conversely, late-onset and milder subtypes show lower genetic contributions (11). At the symptomatic level, clinical evidence suggests that the internal structure of depression is not uniform. For example, melancholic depression typically includes features such as anhedonia, early morning awakening, and psychomotor disturbance, whereas atypical depression includes mood reactivity, hypersomnia, and increased appetite. Such symptom-specific differences imply that some subtypes, particularly severe and suicidal depression, may be more treatment-resistant and require intensive, tailored interventions, while milder or situational forms might respond well to brief or low-intensity treatments (12). The biological correlates and symptom profiles of different depression subtypes are summarized in Table 1. Recent research using both Diagnostic Statistical Manual (DSM)-5 (25, 26) and data-driven methods consistently supports the existence of biologically distinct depression subtypes. Data-driven clustering reveals unique biological signatures, particularly in inflammatory markers and brain connectivity, for symptom groups such as neurovegetative, anhedonic, and anxious profiles (27, 28). However, progress is hindered by methodological variability, limited replication, and inconsistent reporting, underscoring the need for more standardized, multimodal approaches to fully delineate and validate these subtypes. This variability calls for more personalized interventions that address biological correlates in addition to symptom profiles (14, 24).

**Table 1:** Symptom profiles and biological underpinnings of depression subtypes.

Biological correlate	Associated symptoms	Depression subtypes
HPA-Axis Dysfunction Elevated basal cortisol; altered cortisol awakening response (hyper- or hypo-cortisolemia) (13, 14)	Insomnia or early-morning awakening; hyperarousal; anxiety sensitivity; weight/appetite loss or gain.	Melancholic/typical depression (HPA hyperactivity); Atypical/neurovegetative depression (blunted or normal cortisol response)
Inflammatory Markers ↑ CRP, IL-6, TNF-α, IL-8 (12, 13, 15)	Persistent fatigue; hypersomnia; somatic pain; appetite changes; “sickness” malaise	Atypical/neurovegetative depression (elevated inflammation); no elevation in melancholic/typical depression
Metabolic Indicators ↑ BMI, waist circumference, triglycerides, metabolic-syndrome markers (13, 16)	Weight gain; increased appetite; fatigue	Atypical/neurovegetative depression (metabolic dysregulation); no abnormalities in melancholic/typical depression
Neurotransmitter Dysregulation CRH–LC-NE hyperactivity; dopamine-agonist responsiveness (14, 17)	Anhedonia; low motivation; mood instability; irritability; anxiety; psychomotor changes	Melancholic depression (CRH–LC-NE hyperactivity); Atypical depression (dopamine-agonist responsive with reduced CRH–LC-NE tone)
Neural Circuitry Alterations Aberrant connectivity in DMN, salience, and cognitive-control networks (18–20)	Rumination and self-referential thought; psychomotor retardation; apathy; impaired executive function	Anxious-ruminative subtype (DMN hyperconnectivity); Melancholic/severe MDD (DMN hypoconnectivity); plus four data-driven “biotypes” predictive of treatment response
Genetic Risk Profiles Polygenic scores for MDD, schizophrenia, BMI- and metabolic-trait loci (10, 19)	Early-onset/recurrent course; cognitive impairment; diurnal mood variation; metabolic dysregulation	Familial/early-onset depression (high MDD-PRS); MDD with psychotic features (schizophrenia-PRS); Atypical/metabolic subtype (BMI- and triglyceride-PRS)
Kynurenine Pathway Dysregulation ↑ QUIN/KYNA ratio; ↓ kynurenic acid (16, 17)	Cognitive deficits (impaired concentration, memory); increased suicidality risk; anhedonia via serotonin depletion	Neuroinflammatory/cognitive-impairment subtype; TRD with neurotoxic kynurenine signature
BDNF Alterations ↓ Serum BDNF levels (21)	Anhedonia; reduced motivation; impaired synaptic plasticity; memory problems	Neurotrophic-deficit depression; TRD characterized by diminished neuroplasticity
Immune-Cell Changes ↑ WBC, lymphocytes, platelets; altered lymphocyte proliferation (15, 21)	Somatic aches and pains; heightened stress reactivity; poor antidepressant response	Immune-driven/inflammatory depression; TRD with pronounced cellular-immune activation
Hippocampal Alterations Reduced volume, impaired neurogenesis, altered regulation of HPA axis (22)	Contextual memory deficits and impaired pattern separation leading to overgeneralization of negative contexts	Chronic/recurrent MDD (volume loss after ≥2 years or multiple episodes); Stress-related depression (volume loss secondary to acute/chronic stress); Cognitive-impairment subtype characterized by poor memory and learning
Catatonic Syndrome Motor abnormalities (stupor, mutism, posturing, negativism, echophenomena); GABAergic dysregulation in basal ganglia–cortical circuits; right orbitofrontal hypoactivity (23, 24)	Stupor; mutism; negativism; posturing; echolalia/echopraxia; extreme anxiety; contextual overgeneralization	MDD with catatonic features (catatonic subtype)
Postpartum Endocrine & Immune Dysregulation Rapid withdrawal of estradiol/progesterone; reduced oxytocin; HPA-axis hypoactivation; ↑IL-6 and proinflammatory cytokines; serotonergic and BDNF gene polymorphisms (4, 25, 26)	Depressed mood; anhedonia; sleep/appetite disturbances; fatigue; anxiety; cognitive impairment	MDD with peripartum onset (Postpartum depression)

HPA, hypothalamic–pituitary–adrenal axis; CRP, C-reactive protein; IL-6, IL-8, interleukin-6, interleukin-8 (pro-inflammatory cytokines); TNF-α, tumor necrosis factor alpha; BMI, body mass index; CRH, corticotropin-releasing hormone; LC, locus coeruleus; NE, norepinephrine; DMN, default mode network; MDD, major depressive disorder; PRS, polygenic risk score; QUIN, quinolinic acid; KYNA, kynurenic acid; TRD, treatment-resistant depression; BDNF, brain-derived neurotrophic factor; WBC, white blood cells; GABA, gamma-aminobutyric acid.

### 1.2 Depression management in clinical practice

Clinical guidelines such as the DSM-5 and National Institute for Health and Care Excellence (NICE) acknowledge clinical heterogeneity in depression by incorporating specifiers and stratified care approaches rather than redefining novel biological subtypes. The DSM-5 introduces specifiers “with melancholic features”, “with atypical features”, “with anxious distress”, and others to capture distinct symptom patterns that may guide treatment selection—for example, considering electroconvulsive therapy in severe melancholic depression or MAO inhibitors in atypical presentations (25). Similarly, NICE stratifies patients by episode severity (“less severe” vs. “more severe”) and recognizes subgroups such as chronic depression, psychotic depression, and treatment-resistant depression, offering stepped-care algorithms and augmentation strategies tailored to each subgroup (29). By refining diagnostic descriptors and matching intervention intensity and modality to individual clinical profiles, these guidelines operationalize depression's heterogeneity within routine practice.

The diverse manifestations of depression directly impact clinical practice, particularly regarding diagnosis, treatment selection, and long-term management. Despite the well-documented heterogeneity of depression, primary care settings, where most depression cases are initially encountered, often rely on broad diagnostic criteria and generalized treatment guidelines, resulting in inconsistent treatment implementation (30). Only 30% of patients achieve remission with their first prescribed medication, raising the need for multiple treatment trials before finding effective interventions, extending suffering, and increasing healthcare costs. Furthermore, approximately 55% experience side effects, highlighting the urgent need for personalized treatment strategies (8, 16, 20).

Precision psychiatry aims to tailor mental health treatments to groups and ultimately to individual patients (personalized psychiatry) by integrating biological, clinical, and digital data to predict treatment response and optimize therapeutic outcomes. Despite its potential, precision psychiatry remains difficult to implement. A key challenge is the lack of biomarker-based diagnostics. While psychiatric diagnoses are often reproducible, their biological validity remains weak, making precise treatment selection difficult (20). Measurement-Based Care (MBC) can improve treatment outcomes by using validated scales to systematically track symptoms, guide clinical decisions, and enhance patient adherence. Research demonstrates that MBC significantly increases remission rates (74% vs. 29% in standard care) and doubles treatment response odds in primary care settings (31).

### 1.3 Personalizing depression care

Recognition of depression's heterogeneous nature has driven momentum toward personalized medicine approaches that tailor treatment strategies to individual patient characteristics. These personalization strategies incorporate biological, psychological, and digital tools to refine diagnosis, predict treatment response, and improve outcomes as detailed below.

#### 1.3.1 Pharmacogenetics and biomarkers

Pharmacogenetic testing examines how genetic variations influence individual responses to antidepressants (32). Evidence suggests that genetic markers affecting serotonin metabolism (e.g., SLC6A4 polymorphisms) and liver enzyme activity (e.g., CYP450 variants) can predict treatment efficacy and side effects, enabling more informed medication selection. Despite the promise, pharmacogenetics has not yet achieved widespread implementation, as questions regarding cost-effectiveness and clinical utility persist (33).

Biomarkers play a crucial role in advancing psychiatry by aiding in the diagnosis, treatment, and potential prevention of major psychiatric disorders such as depression, schizophrenia, and anxiety. Central (brain imaging) and peripheral (blood proteins, immune markers) biomarkers provide biological signatures that help distinguish between disorders and predict treatment responses (21). On the other hand, digital biomarkers, derived from mobile apps, wearables, and other digital health technologies, are transforming mental healthcare by enabling real-time, objective monitoring of mental states. These biomarkers include speech patterns, sleep metrics, heart rate variability (HRV), activity levels, and human-device interactions, which can provide continuous, passive assessment of mental health conditions (34, 35).

#### 1.3.2 Personality subtypes and psychological stratification

Recent investigations suggest personality traits play a key role in depression subtyping and treatment optimization (36, 37). Personality traits like high neuroticism, low extraversion, and low conscientiousness are associated with greater responsiveness to intensive interventions such as CBT (38). Furthermore, high levels of self-criticism, social avoidance, and personal reserve predict poorer responses to psychological therapies, underscoring the importance of tailored care not only to biological profile but also to psychological characteristics of patients (39, 40). Stratified care models—where treatments are assigned based on initial psychological assessments—have demonstrated greater clinical and cost-effectiveness compared to traditional stepped care approaches (23).

#### 1.3.3 Digital phenotyping and machine learning (ML)

Digital phenotyping, which refers to the real-time and passive collection of behavioral data through digital devices, offers an unprecedented opportunity to understand the nuanced, moment-to-moment patterns of mental illness. Smartphones and wearables can unobtrusively track digital biomarkers, creating a digital fingerprint of each individual's mental state. These data, when paired with ML algorithms, enable the identification of personalized behavioral signatures linked to psychiatric conditions and treatment responses (41, 42). Algorithms trained on vast multimodal datasets—including active inputs like ecological momentary assessments (EMAs) and passive data from sensors—can predict depressive episodes, monitor treatment efficacy, and match patients to the most effective therapeutic modalities (42). Wearable devices gather continuous physiological and activity signals [e.g., heart rate (HR), movement, sleep patterns], which can be processed into metrics like resting heart rate or sleep efficiency. EMAs involve time-stamped, real-time self-reports via smartphones on mood, stress, context, and behaviors. Together, wearables offer objective sensor data, while EMA provides subjective, contextual insights (43).

### 1.4 Objectives of the review

This is perhaps the first review to discuss the heterogeneity of depression in relation to digital health. This systematic review aims to explore and analyze recent digital technology targeting personalized depression care, thereby making the case for the digital transformation of mental healthcare. The objectives are:

a. To summarize and critique the recent literature on personalized digital interventions.
b. To critically assess how personalized digital health interventions address the heterogeneity of depression
c. To identify key challenges and future directions in the integration of digital health into personalized mental health care.

## 2 Methods

This paper adopts a systematic review approach to critically synthesize and evaluate the current literature on the role of digital health interventions in addressing the clinical heterogeneity of depression.

### 2.1 Search strategy

**Figure 2:**
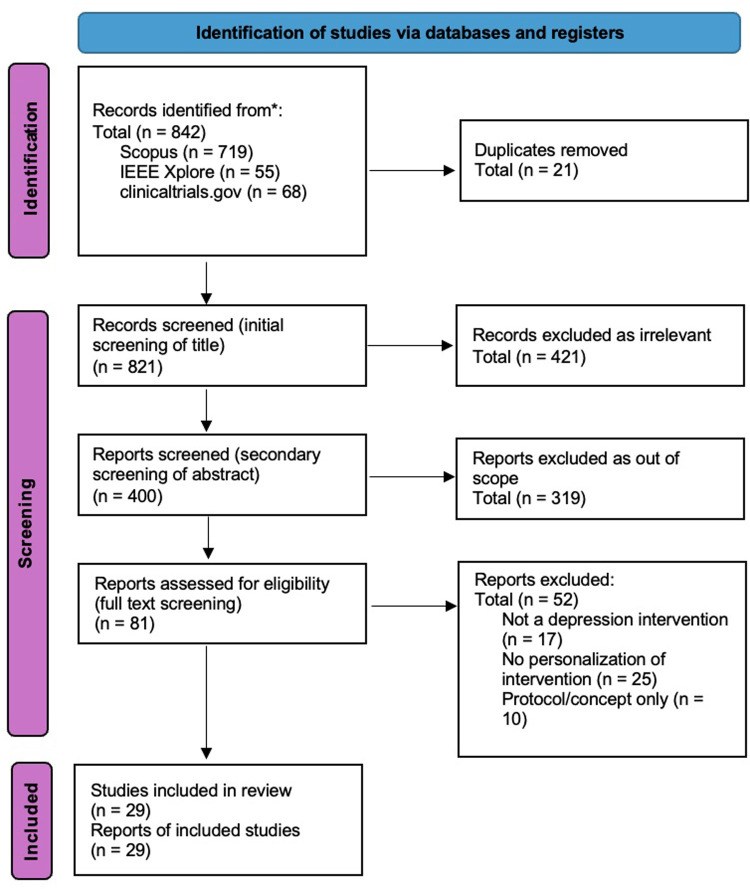
PRISMA diagram of literature from January 2020 to September 2025.

This review focuses on a specific niche in depression care: the personalization of digital health solutions to target the biological correlates of depression subtypes. While much research has explored either depression heterogeneity or digital tools independently, few have bridged both areas. The search strategy targeting this specific niche is shown in Table 2. This review intentionally focused on personalized digital interventions for depression; studies without an explicit personalization component were excluded by design. Searches covered peer-reviewed literature in Scopus and IEEE Xplore, complemented by targeted grey literature from ClinicalTrials.gov and hand-searching of reference lists and forward citations. Searches were last run on 15 September 2025. Records published after this date were not considered. Following the PRISMA guidelines, 29 studies were identified that address heterogeneity of depression to varying levels: 20 papers and 9 clinical trial reports. The PRISMA scheme is shown in Figure 2.

**Table 2:** Search strategy.

Databases searched	Scopus, IEEE Xplore, ClinicalTrials.gov
Search keywords	TITLE-ABS-KEY [(“Mental Health” OR “Depression” OR “Mood Disorders”) AND (“Digital Health” OR “mHealth” OR “eHealth” OR “Mobile Mental Health Apps” OR “Digital intervention” OR “internet-delivered”) AND (“personalized” OR “individualized” OR “patient-specific” OR “Adaptive”)] AND PUBYEAR>2019 AND PUBYEAR<2026 AND [LIMIT-TO (DOCTYPE, “ar”) OR LIMIT-TO (DOCTYPE, “cp”)] In ClinicalTrials.gov, the search was for Major Depressive Disorder, other terms are Depression and Digital.
Search period	From January 2020 and September 2025, focusing on recent advancements in digital health and personalized psychiatry.
Language	Only articles published in English were included.
Inclusion criteria	1. Peer-reviewed research articles, randomized controlled trials, observational studies, and conference papers relevant to depression and digital health. 2. Studies focusing specifically on depression and depressive symptoms. 3. Studies presenting a digital intervention or mHealth tool with explicit discussion of personalization technique.
Exclusion criteria	1. Review papers, concept papers, proposals, and theoretical frameworks, design studies or studies assessing the design of intevrention rather than the outcome. 2. Studies focusing on other disorders or general mental health and wellbeing without a focus on depression. 3. Studies unrelated to a digital interventions. 4. Studies focusing solely on generic digital health tools without a personalized or adaptive component.

### 2.2 Data extraction and synthesis

Data was extracted using Microsoft Excel by a single author, G.A. The extracted information included research objectives, methods, personalization strategy, key findings, and other study details. Due to the heterogeneous nature of the studies, the data extraction and synthesis follow a narrative, descriptive approach organized along two orthogonal frameworks: (i) modality of intervention (passive sensing/forecasting; just-in-time adaptive interventions; conversational/coach-supported tools; decision-support/recommenders; physiology-coupled therapeutics) and (ii) personalization axis (timing & context, treatment format & dose, content & target). Data items include:

- Bibliographic and Design Details: author(s), year, country, study design (e.g., randomized controlled trials, cohort, case series).
- Participants: sample size, clinical population (e.g., MDD diagnosis, subclinical), age range, and demographic composition.
- Intervention Characteristics: digital modality (e.g., smartphone app, wearable), personalization strategy (e.g., speaker-specific layers, transfer learning, co-design), duration, and comparator.
- Outcomes and Measures: primary and secondary outcomes (e.g., PHQ-2/9 scores, mood correlation coefficients, Cohen's d, prediction accuracy), assessment instruments, and follow-up intervals.
- Implementation and Contextual Variables: technology platform, engagement metrics (e.g., retention rates), and any human-centred design or equity considerations reported.

### 2.3 SWOT analysis

To systematically evaluate the empirical literature on personalized digital interventions for depression, SWOT (Strengths, Weaknesses, Opportunities, Threats) analysis was employed. This strategic framework is commonly used in healthcare research to critically assess innovation potential, implementation challenges, and contextual fit. In this review, SWOT was applied to 20 peer-reviewed studies, each offering a unique technological or methodological approach to personalized mental health care.

### 2.4 Risk of bias assessment

To evaluate the methodological rigor and reliability of 14 prospective studies, a qualitative Risk of Bias (RoB) (44) assessment was conducted. This process considers five core domains adapted from the Cochrane Risk of Bias framework and digital intervention research best practices. Each study was categorized as Low, Moderate, or High risk in each domain, leading to an overall risk of bias judgment. This qualitative appraisal supported a nuanced interpretation of findings across heterogeneous methodologies.

The Prediction Model Risk Of Bias Assessment Tool (PROBAST) (45) was used to assess the quality of the models used in the 3 modeling studies. The models were evaluated for signal problems in the four domains of PROBAST, and the results were categorized as “yes”, “probably yes”, “probably not”, “no”, or “no information”. This leaves 3 articles (13, 46, 47) that were not fit for these tools and were narratively appraised instead. Only one researcher, G.A., performed the risk of bias assessments.

## 3 Results

The development of personalized digital interventions is transforming depression care by incorporating user-specific data and adaptive technologies into treatment delivery. Recent empirical studies demonstrate that personalization can significantly enhance mental health outcomes.

One promising avenue is real-time monitoring and forecasting using passive mobile sensing and EMAs to improve symptom prediction. Individualized models consistently outperform pooled approaches. For example, ML models trained on individual-level mobile data improved depression forecasting accuracy by up to 25% over non-personalized models (48). Similarly, personalized deep learning models using speech features outperformed general models in predicting mood states, highlighting the value of speaker-specific adaptations (17). In another study leveraging wearable and EMA data, Chatterjee et al. (49) developed explainable deep learning models that predicted mood scores with as little as 6% error for some participants. Their approach not only enabled high accuracy but also facilitated insight into which biophysical indicators contributed to mood changes, underscoring the potential of explainable, individualized systems to support tailored interventions. However, both studies are limited by small sample sizes and missing data. Digital monitoring of depressive symptoms in older adults was studied using a smartwatch, a motion-sesing camera, and a chatbot. This living-lab platform gives them daily, baseline-anchored updates on their mental and physical health to support self-care, and strengthens social support by sharing daily status and emergency alerts with community caregivers (50).

Personalized support delivered “in the moment” demonstrates additional benefits. An automated, personalized smartphone program targeting repetitive negative was tested in a pilot randomized trial. The Mello app, a just-in-time adaptive intervention (JITAI), outperformed controls, reducing repetitive negative thinking, anxiety, and depressive symptoms (51). Another rumination-focused intervention, by Wang and Miller (52), pilot-tested a fully automated JITAI using CBT to identify and block depressive rumination. They used EMAs to drive a just-in-time system where every few hours participants reported recent stressors and, if a rumination trigger was present, the app identified the trigger type and checked whether the person was currently receptive (e.g., not driving or walking), then delivered tailored support. The study participants reported reduced episode counts and duration when support was triggered just after rumination was detected. Future validation with a larger sample size is essential (52). Cue, a precision smartphone program for outpatient care improved symptoms by timing small interventions to each person's daily routines or “social rhythms” such as sleep timing, daily mood, and energy self-ratings. Cue is a smartphone-based platform that continuously tracks behavior and collects self-reports. It pairs psychoeducation modules with personalized “micro-interventions”; very short, targeted therapeutic actions, usually seconds to a few minutes, delivered in the flow of daily life, for example, reminders to keep a consistent wake time if weekend sleep-ins are detected. The aim is to regularize daily routines and, in turn, reduce depressive symptoms and sustain wellness with minimal patient effort (53). Another JITAI, delivered via m-Path smartphone app, was to designed to provide psychological support to individuals experiencing subclinical and clinical levels of depressive symptoms while awaiting psychotherapy. Once triggered, the intervention asks participants to name the kind of social support they require, or surfaces a list of past contacts to identify who could help right now, or delivers one of six evidence-based support-seeking strategies. Participants then choose how to reach out. Microrandomization is performed at each eligible decision point, where the participant was found in need of support, they are randomized to either intervention or control. Microrandomized feasibility work showed high adherence, small immediate distress reductions, and the highest perceived appropriateness when individuals themselves signaled need in real time (19).

Communication-centered supports, ranging from smart messaging to agent-guided use, provide lower-intensity personalization pathways that can scaffold care. A novel study by Malins et al. (54) applied personalized “smart-messaging” to support CBT follow-up care. Participants prewrote advice tailored to their future emotional states, which was delivered via text after therapy ended. Over 12 months, users of this personalized system showed greater improvements and stability in well-being than non-users, suggesting that low-intensity digital personalization can meaningfully support relapse prevention in clinical populations (54). While there is a lot of research on chatbots targeting mental health support, few offer personalized theraputic support to depressed populations. Woebot® (WB001) is a prescription, 8-week mobile digital therapeutic used for postpartum depression, among other mental health conditions, that uses a conversational agent to deliver brief, personalized CBT, daily mood tracking, and psychoeducation under clinician oversight. It tailors chats to user-selected problem areas, maintains rapport via Natural Language Processing (NLP), and includes crisis detection with SOS escalation. Users show symptom reduction and high satisfaction, with evidence of therapeutic alliance and preliminary efficacy in young adults and postpartum samples (13). Another chatbot, MindBot, powered by AI, delivers personalized mental-health support by combining classic NLP like tokenization and sentiment scoring with large language models (LLMs) for context-aware replies. Bench testing showed it can track shifting emotions, handle typos, and maintain stable performance. However, evaluation focused on accuracy, “emotional accuracy”, and reliability, not clinical outcomes (46).

While this review is not focused on clinician-guided personalization of care, as that has been addressed in previous literature and is out of scope, certain studies have incorporated human-guided personalization with algorithmic tailoring of therapeutic content. For example, HAYT (“How Are You Today?”) is a mobile app for anxiety and depression that allows patients to directly chat with clinicans instead of chatbots. It combines a suite of services and features: a digital diary analyzed with NLP to detect sentiment and symptoms; DSM-5–derived symptom questionnaires; a predictive model for near-term risk; personalized CBT prompts; secure messaging/video with clinicians; scheduling/reminders; and clinician-facing reports. The preliminary results, though based on synthetic data, indicate a strong correlation between sentiment analysis and self-reported depressive symptoms, suggesting its utility in monitoring mental health in a clinical setting (47). Another app, mindLAMP was used to test the effect of having perosnalized recommendations given by a Digital Navigator “Guide” vs. general untailored content 'Support'. A Digital Navigator is a care-team specialist in digital health who supports patients with technical troubleshooting, tailors and optimizes app use to boost engagement, and helps integrate digital tools into clinical care. Both groups had full access to the mindLAMP app, which included modules like Thought Patterns, Mindfulness, Journaling, Distraction Games, Gratitude Journaling, Behavioral Activation, and Strengths. Guide users completed significantly more activities overall (*p* < .001), while Support users “binged” early then tapered. Notably, even though the two coaching groups didn't differ statistically, the people who used the app more were more likely to get big improvements; participants with ≥25% drops in PHQ-9/GAD-7 had higher Digital Working Alliance Inventory scores than non-improvers (55).

Decision rules and recommender logic translate personalization into actionable choices about “what to do” or “what level of care to provide”. A data-driven, personalized activity recommender for mood disorders learns which specific activities boost an individual's mood. Data was gathered over several weeks from two mobile apps spanning clinical and non-clinical populations. In the MORIBUS clinical sample, 7 patients with unipolar or bipolar disorder logged 1,684 entries, selecting or typing specific activities and rating each activity's positivity. Text and labels from activity logs are preprocessed and modeled with Naive Bayes and SVM, comparing a pooled (“general”) vs. person-specific model. After roughly 59 activities per user, personalized models significantly outperform general ones with error rates as low as 10% for some participants (56). Another recommender, the Personalised and Optimised Therapy algorithm, trained on the 4,469 participants in the RESiLIENT trial using regularized prediction models and early Patient Health Questionnaire-9 response. The aims is to estimate individual treatment effects across cognitive behavioral therapy skills and select the option with the highest probability of benefit. It recommends single skills for very low baseline severity and two-skill combinations for higher severity. In a simulated trial, it significantly increased overall treatment effect with approximately 35% greater benefit than the non-personalized group average (15).

Measurement-based, stratified systems of care that adjust level and type of support to ongoing assessments offer personalization in the clinical practice. STAND is a stepped-care model for university students that screens with the Computer Adaptive Test for Mental Health, assigns a care tier (T0-monitoring only, T1-digital therapy with coaches, T2-digital therapy assisted by clinicians in training, and T3-clinical care), and adapts level based on continuous symptom and suicide-risk monitoring. In deployment, hundreds of suicide-risk alerts were detected and managed in real time. Acceptability was high across tiers. Depression and anxiety improved significantly (*P* < .001) in all tiers and engaged participants showed ≥30% symptom reductions (57).

Finally, physiology-coupled and lifestyle-tailored therapeutics personalize timing and content using signals beyond self-report. An e-health program, NEVERMIND, combined a sensorized smart shirt (electrocardiogram, respiration, movement) with a mobile app that administers questionnaires, forecasts depressive symptoms, and delivers personalized feedback and lifestyle guidance, and online cognitive behavioral therapy via Deprexis. The program significantly reduced depressive symptoms and suicidal ideation compared to control (*p* < 0·001), with a clinically relevant effect size (Cohen's *d* = 0·39) (58). In active-duty personnel, a randomized study in military personnel tested CBT alone vs. CBT plus a smartwatch-linked mHealth app that detects physiological stress, delivers real-time alerts with guided coping, and shares data with therapists via a provider portal. The app group attended more sessions and showed significant reductions in depression, anxiety, stress, and anger, approaching asymptomatic levels by approximately 45 days (59). Another randomized waitlist-controlled trial tested FeelDTx. By integrating a mobile CBT-based program, a wearable emotion sensor (EDA, HRV, skin temperature) that triggers personalized, in-the-moment prompts, and weekly 15-minute Digital Navigator check-ins, the study reported reported high engagement and larger symptom reductions than the control. In the experimental symptomatic group, depressive and anxiety symptoms fell by 45% and 50% from baseline, respectively, with 32 and 39 subjects exceeding the Minimal Clinically Important Difference, respectively (60). In a unique approach to managing depressive symptoms, Campisi et al. (61) conducted an 8-week single-arm pilot of a personalized nutrition program for adolescents with MDD. They employed bi-weekly counseling with a Mediterranean-style co-created menu, as well as weekly groceries and eHealth messages (61). For a small sample of 10 parent-teenager pairs, feasibility was moderate (40% recruitment; 77% completion) with moderate–high acceptability. Preliminary effects showed small improvements in depressive symptoms (*d* ≈ 0.36), parent food modeling (*d* ≈ 0.24), and adolescent nutrition attitudes (*d* ≈ 0.36) (61).

Taken together, the evidence supports a thematic map in which real-time monitoring and forecasting quantify within-person dynamics; just-in-time support and messaging deliver timely, symptom-oriented help; agents and human coaches personalize communication and adherence; decision rules and recommenders choose content or level of care; and physiology- or lifestyle-coupled therapeutics align delivery to physiological state and daily context. Across these modalities, personalization functions as the unifying mechanism most closely associated with improved engagement and clinical improvement. The details of these studies are summarized in Table 3, which organizes results by modality and adaptivity to clarify similarities and differences across approaches.

**Table 3:** Summary of reviewed papers grouped by modality.

Study; sample size; design	Personalization axis	Data sources used	Target mechanism or phenotype	Adaptivity	Primary outcome and effect metric (as reported)
A. Passive sensing and forecasting: Learns within-person patterns from ambient data; output is prediction/monitoring, not necessarily an intervention.					
(48) — Personalized depression forecasting using mobile sensor data and ecological momentary assessment; *N* = 65; observational modeling within a digital intervention cohort	Model-based (subject-dependent standardization; transfer learning; subgroup models)	Ecological momentary assessments, smartphone sensors	Depressive symptom severity prediction and next-day symptom forecasting	Static tailoring (modeling; not a real-time intervention)	End-of-day Patient Health Questionnaire-2 mean absolute error 0.801 (approximately 25% better than baseline 1.062); next-day mean absolute error 1.349 (approximately 12% better than baseline 1.539)
(49) — Towards personalized mood prediction and explanation for depression from biophysical data; *N* = 14; observational modeling	Model-based deep learning with model explainability	Ecological momentary assessments, wearable lifestyle data, neurocognitive assessments	Current mood state and depressive symptom severity	Static tailoring (offline personalized models)	Per-person prediction error reported as low as approximately six percent; deep learning models exceeded classical machine-learning baselines
(17) — Personalized deep learning for monitoring depressed mood from speech; *N* = 41 (30 major depressive disorder and 11 subclinical); observational modeling	Model-based	Speech captured via smartphone during ecological momentary assessment sessions	Depressed mood monitoring on a visual analogue mood scale	Static tailoring (offline personalized models)	Personalized models outperformed population models; higher correlation between predicted and self-reported mood (details in Supplement)
(50) — Socially vulnerable older adults; six-week single-arm living-lab pilot; *N* = 25	Rule-based individualized daily feedback dashboards	Daily Patient Health Questionnaire assessments via chatbot; wearable sensor data including heart rate variability, sleep, and physical activity	Day-to-day depressive symptom fluctuations in geriatric populations	Static daily feedback without just-in-time logic	Within-person modeling showed sleep fragmentation and sleep efficiency associated with higher same-day depressive symptoms; pre–post improvement in depressive symptoms and sleep quality; usability unchanged
B. Just-in-time digital support: Automated micro-interventions delivered at high-value moments, often driven by ecological momentary assessment or sensors.					
(51) — A personalized, transdiagnostic smartphone intervention (Mello) targeting repetitive negative thinking; *N* = 55; pilot randomized controlled trial	Model-based adaptive intervention (fully automated and personalized)	Ecological momentary assessments via smartphone	Repetitive negative thinking as a transdiagnostic mechanism; depression and anxiety symptoms	Adaptive in real time (just-in-time delivery)	Depression standardized effect size approximately 0.50; anxiety standardized effect size approximately 0.61; repetitive negative thinking standardized effect size approximately 0.87 over twelve weeks
(53) — Social rhythm–focused precision digital intervention (Cue) augmenting outpatient care; intent-to-treat *N* = 133; depressed-at-entry subgroup *N* = 28	Model-based personalization using smartphone behavior to time micro-interventions	Continuous smartphone behavioral patterns; symptom self-reports	Repetitive negative thinking and depressive symptoms with anxiety symptoms as secondary outcomes	Adaptive in real time with just-in-time smartphone delivery	Greater improvement from baseline to sixteen weeks in the full sample; larger reduction in Patient Health Questionnaire-8 scores in depressed-at-entry subgroup versus monitoring only
(52) — Pilot randomized controlled trial of rumination-focused mobile cognitive behavioral therapy just-in-time adaptive intervention; *N* = 18	Personalized timing using each participant's rumination pattern	Intensive self-reports via smartphone text messages; mobile intervention content	Depressive rumination episodes, duration, and carryover	Just-in-time adaptive delivery after detected rumination	Greater reductions in rumination episodes and minutes ruminating; evidence of reduced rumination carryover versus control
(19) — Social support just-in-time adaptive intervention while awaiting psychotherapy; microrandomized feasibility study; *N* = 25	Rule-based and personalized decision rules including fixed cutoffs, Shewhart control charts, or self-reported need	High-frequency ecological momentary assessments of negative affect, stress, loneliness, and rumination; in-application prompts and support-seeking logs	Distress reduction by mobilizing social support during vulnerable moments	Adaptive in real time; intervention triggered by real-time assessments according to microrandomized decision rules	High feasibility and compliance; interventions triggered by self-reported need were rated most appropriate and helpful; exploratory distress reductions with small effect sizes
C. Smart messaging and convesational agents: Scheduled or light-tailoring text/app messages that maintain gains or nudge behavior, Agent or therapist-guided online CBT or supportive chat.					
(54) — Smart-messaging as relapse prevention following psychological therapy; observational cohort in routine care; [Study 1: 53 out of 79 completed CBT, divided into smart-messaging (15) and no-messaging (38) groups. Study 2: 14 participants used smart-messaging.]	Rule-based tailoring	Short message service check-ins and follow-up symptom measures	Symptom improvement and relapse prevention after therapy	Static delivery (scheduled tailored messages; not real-time adaptive)	Greater twelve-month symptom improvement for smart-messaging users compared with non-users; stability at six months in routine practice
(13) — Woebot WB001 for postpartum depression; device profile and synthesis of efficacy signals; *N* = 36,070	Model-guided and agent-guided tailoring	In-application conversational exchanges; measurement-based care elements	Postpartum depression symptoms and interpersonal stressors	Adaptive conversational guidance	Device profile summarizing design and supportive efficacy data; no single definitive randomized outcome reported in this article
(46) — MindBot conversational agent (engineering and evaluation report, not tested with users)	Model-based personalization using real-time sentiment monitoring and large language models	In-application conversations; sentiment analysis; predefined templates and dynamic responses	Depressive sentiments and supportive engagement	Adaptive conversational responses	Usability and engagement-oriented results; no clinical depression outcomes reported in this paper
(47) — “How Are You Today?” mobile application using natural language processing to support diagnosis and treatment of anxiety and depression; feasibility engineering report (*N* = 63 synthetic diary entries of a single depressed subject over a nine-week period)	Model-based natural language processing with clinician-tailored follow up	Free-text diary entries; diagnostic screening based on the Diagnostic and Statistical Manual of Mental Disorders Fifth Edition; in-application interactions and notifications	Depressive and anxiety symptom monitoring and prediction of anxiety or panic episodes	Adaptive prompts and feedback based on language analysis with clinician escalation available	Preliminary feasibility with synthetic data; correlation between diary sentiment analysis and self-reported depressive symptom scores; no randomized clinical outcomes reported
(55) — Digital Navigator coaching: guided versus supportive models with mindLAMP; *N* = 156; six-week comparative study	Clinician-guided personalization of application recommendations (Digital Navigator “Guide” versus “Support”)	Smartphone application use logs, activities completed; survey outcomes	Depression and anxiety symptoms; engagement with therapeutic activities	Human-guided adaptation over time with scheduled contacts and tailored suggestions	Guide group completed more activities; thirty-four percent showed at least twenty-five percent decrease in Patient Health Questionnaire-9; thirty-eight percent showed at least twenty-five percent decrease in Generalized Anxiety Disorder-7 overall
D. Decision-support and recommenders: Personalized suggestions or level-of-care decisions learned from responses and preferences, Algorithms choose the best skills/modules for a person.					
(56) — Recommending activities for mental health and well-being: insights from two user studies; a clinical sample (*N* = 318 activities/user) and a non-clinical sample (*N* = 59 activities/user).; observational modeling	Model-based	Ecological momentary assessment activity logs and ratings	Behavioral activation target through positive-affect activities	Static tailoring (offline recommendations; not just-in-time)	Personalized models outperformed pooled models; approximately fifty-nine activities per user required before personalized models surpassed general models
(15) — Personalized and optimized therapy algorithm for subthreshold depression (RESiLIENT trial); randomized smartphone cognitive behavioral therapy with prescriptive modeling; *N* = 4,469	Model-based prescriptive algorithm recommending best skill or combination	In-application Patient Health Questionnaire-9 and usage data	Depressive symptom reduction in subthreshold depression	Adaptive selection at assignment stage (not continuous just-in-time)	Simulated randomized comparison: personalized and optimized therapy outperformed health information control with standardized mean difference approximately −0.37 and approximately thirty-five percent greater benefit than group-average best
(57) — Screening and Treatment for Anxiety and Depression; *N* = 516 treated from 5,000 screened; open trial in a university system	Rule-based triage and adaptation by symptom severity and suicide risk	Computerized adaptive testing delivered remotely; ongoing symptom monitoring	Depression and anxiety symptom burden; suicide risk	Dynamic adaptation of level of care over forty weeks	Significant symptom improvements across tiers; feasibility and acceptability reported (no randomized comparison)
E. Wearable-integration and lifestyle support digital interventions: Apps tightly coupled with physiology to personalize timing/content, Tailoring lifestyle inputs as part of a digital care plan.					
(61) — Personalized nutrition for adolescent major depressive disorder; *N* = 10; single-arm mixed-methods feasibility	Clinician-tailored menus, stepped dietary goals, family context tailoring	Virtual counseling sessions, menu plans, grocery delivery, educational electronic health messages	Depressive symptoms via dietary-mechanism change; family food environment	Scheduled step-up across four bi-weekly sessions	Feasibility achieved; depressive symptoms improved with small-to-moderate effect (Cohen's d approximately 0.36; wide confidence interval)
(60) — Randomized controlled study of a digital data-driven therapeutic for depressive and generalized anxiety symptoms; *N* = 200 randomized; sixteen weeks	Data-driven personalization of intervention timing and content	Wearable physiology such as skin conductance, activity, and sleep; mobile application interactions	Depressive and generalized anxiety symptoms	Data-triggered adaptive delivery throughout treatment	Intervention achieved larger reductions than waitlist control with high engagement reported
(58) — NEVERMIND pragmatic randomized controlled trial; *N* = 425	Personalized behavioral content within application modules	Wearable physiological data via smart shirt; mobile application interactions; questionnaires	Depressive symptoms among patients with severe somatic conditions	Scheduled content; not just-in-time	Lower depressive symptoms at twelve weeks versus standard care; effect maintained in per-protocol analysis
(59) — Randomized controlled trial in military personnel (*N* = 30, divided over three arms)	Data-triggered personalized guidance and provider communication	Wearable physiology; mobile application stress alerts; symptom measures	Symptoms of depression, anxiety, stress, and anger in active-duty populations	Real-time alerts prompting immediate coping techniques	Application group completed therapy and showed significant symptom reductions; control cognitive behavioral therapy group had high dropout

Across registered clinical trials and device studies (summarized in Table 4), personalization is being operationalized along three complementary axes: (i) timing and context; detecting when an individual most needs support and delivering it in the moment, (ii) treatment format and dose; adapting the level of human support or modality based on early response, and (iii) content and target; matching therapeutic ingredients to individual symptom mechanisms, cognitive profiles, or neurobiological signals. Together, these trials test whether precision in *when*, *how*, and *what* is delivered can improve outcomes, adherence, and scalability across routine-care and home settings. While peer-reviewed outcomes for many of these trials are pending, their methodologies and design indicate a significant move toward addressing the complexity of depression at the individual level.

**Table 4:** Summary of personalized depression digital health clinical trials.

Title	Population	Intervention Type	Addressing Heterogeneity	Personalization Approach
Motor Activity–Subjective Energy (MASE) Project (NCT07059234)	Adults with major depressive disorder (*N* = 180)	Just-in-time, state-contingent micro-activity prescription informed by within-person activity–energy associations and neurobiological profiling	High (integrates timing and context with neurobiological phenotyping)	Learns individual activity–energy coupling from accelerometry and ecological diaries; applies brain network analyses to stratify who benefits from which micro-activity; delivers when/where/what prompts in daily life
Digital neurotherapy with REJUVENATE (NCT04961047)	Cancer survivors and adults with end-stage kidney disease on dialysis with depressive symptoms (*N* = 36)	Mechanism-targeted digital neurotherapy that adapts cognitive task difficulty across attention, inhibition, working memory, flexibility, processing speed, pattern recognition, categorization, and multitasking	High (content and target tailored to cognitive phenotype; neuroplasticity rationale)	In-app telemetry drives individualized progression rules; session-by-session calibration of task parameters to the participant's evolving cognitive profile
MEL-T01 “Meliora” game-based digital therapeutics (NCT05426265)	Adults with major depressive disorder (*N* = 1,001)	Personalized cognitive training embedded in gameplay with continuous adaptation to executive function performance	High (content and target tailored to neurocognitive profile)	Real-time performance monitoring adjusts task difficulty, stimulus characteristics, and progression schedules to maintain individualized challenge and target executive-control deficits
Targeting network dysfunction in apathy of late-life depression (NCT05877885)	Older adults with late-life depression and clinically significant apathy (*N* = 84)	Customized cognitive-control training targeting attention, salience detection, and cognitive control networks	High (content and target aligned to a neurobiological subtype: apathy)	Training tasks and schedules are tuned to engage hypothesized neural circuits; clinical monitoring supports adherence and safety; seeks circuit-level remediation of apathy-related dysfunction
MIRAI trial of CT-152 (Rejoyn) mobile prescription digital therapeutics (NCT04770285)	Adults with major depressive disorder on stable antidepressant therapy (*N* = 386)	Mechanism-based digital therapeutics that train networks integrating emotion recognition/processing with cognition; measurement-based progression	High (content and target grounded in neuroplasticity and circuit integration)	Structured neurobehavioral exercises progress according to performance and symptom feedback to promote adaptive re-weighting of emotion–cognition circuitry
Circadian Rhythm for Mood (CRM) mobile application (NCT05400785)	Adults with a history of mood episodes (*N* = 93)	Just-in-time relapse prevention via daily mood prediction and personalized alerts	Moderate (timing and context; behavioral signals only)	Personalized next-day risk estimation from wearable activity and daily symptom entries; triggers user-specific prevention guidance when predicted risk exceeds threshold
Personalized, response-based transdiagnostic internet intervention (NCT07051148)	Adults with clinically significant anxiety and/or depressive symptoms (*N* = 366)	Response-adaptive format and dose (self-applied program versus hybrid program with brief therapist sessions for late responders)	Moderate (treatment format and dose adaptation)	Uses early symptom trajectory to classify early versus late responders; late responders randomized to add synchronous therapist sessions; early responders continue or discontinue per protocol
mHELP: Interactive mobile health for high anxiety and depression in college students (NCT07017569)	University students with elevated anxiety, stress, or depressive symptoms (*N* = 125)	Just-in-time support linked to machine-learning stress detection; adjunct telehealth encounters	Moderate (timing and context with minimal clinician input)	Watch and phone signals detect physiologic or behavioral stress; triggers real-time coping tasks and on-demand skills; two scheduled telehealth sessions used as light-touch dose adaptation
MENTINA: Effect of digital markers in self-management of depressive symptoms (NCT06919133)	Adults with current or prior depressive episodes or elevated depressive symptoms (*N* = 660)	Rule-based self-management with escalation: questionnaire and sensor monitoring drive feedback and safety suggestions	Low (content and target via rule-based personalization; safety escalation)	Predefined feedback rules map self-reports and sensor patterns to tailored psychoeducational content and recommendations, including prompts to contact emergency care when indicated

Using timing and context for personalization, the Motor Activity–Subjective Energy (MASE) Project (NCT07059234) learns each person's within-day association between incidental, non-exercise activity and felt energy using accelerometry and ecological diaries, then uses neurobiological profiling (from brain images) to identify who benefits from which micro-activities. The goal is a smartphone system that prescribes “what/when/where” brief activities to raise energy, reduce depressive symptoms, and prevent relapse (62). In a similar paradigm, the CRM mobile application (NCT05400785) combines a wearable activity tracker with daily symptom check-ins and provides personalized mood predictions and prevention prompts to the active arm. The aim is to prevent recurrence by turning forecasts into targeted self-management guidance (63). The mHELP study (NCT07017569) extends real-time adaptation to a campus setting. Students use a watch and phone app for 10 weeks with machine-learning stress detection, on-demand skills (breathing, journaling, media), and two telehealth sessions. The trial tests whether state-contingent prompts and light clinician touchpoints improve anxiety, depression, stress, engagement, and service uptake relative to a monitoring-only control (64).

Following a response-guided format, an adaptive, transdiagnostic internet trial (NCT07051148) begins all participants on a 12-module self-applied program then classifies early vs. late responders after three modules. The program personalizes the format oof support by randomizing late responders to add brief therapist sessions or continue self-guided care. Outcomes include symptom change, emotion regulation, and alliance, directly testing whether early-trajectory signals can right-size human support (65).

Multiple programs tailor the training material to individual cognitive or neural profiles. Yale's feasibility and efficacy study of digital neurotherapy (NCT04961047) delivers eight weeks of personalized cognitive exercises to cancer survivors and patients on dialysis, using the REJUVENATE™ system (66). REJUVENATE™ is an at-home, adaptive digital neurotherapy delivering seven game-based exercises that train attention, inhibition, working memory, cognitive flexibility, processing speed, pattern recognition, categorization, and multitasking (67). Earlier studies of the system showed promising results, neuroplasticity-based computerized cognitive remediation (nCCR) produced greater improvements than control in depression severity and cognition, most notably executive function and verbal fluency (68). Concurrently, brain networks showed restoration toward a more efficient, hub-centric architecture: increased rich-club connectivity (69). Aalto University's MEL-T01 game-based therapeutic (NCT05426265) embedds neurocognitive training and therapeutic content into game mechanics. The main theraputic component consisted of continuous in-game performance measurement coupled with adjustment of the neurocognitive training content to individual executive function levels, which also dynamically change over time. Adults with major depressive disorder are randomized to the active device, a comparator game, or treatment-as-usual, with symptom and cognition outcomes at 4, 8, 12, and 24 weeks (70, 71). Another trial by AdventHealth in late-life depression with apathy (NCT05877885) targets network dysfunction using a customized cognitive-training protocol on the Posit Science platform, with weekly care-manager support. Primary aims include changes in brain connectivity, apathy severity, and cognitive control (72).

Additionally, the MIRAI trial (NCT04770285) evaluates the mobile digital therapeutic CT-152, known as Rejoyn, in adults with major depressive disorder on antidepressant monotherapy. Rejoyn is a prescription app-based digital therapeutic designed to leverage neuroplasticity by training networks that integrate emotion recognition and processing with cognition (73). Data showed consistent symptom improvements across clinician- and patient-reported scales with continued gains one month post-treatment (74). Finally, The MENTINA trial is an international, multicenter randomized controlled trial (Denmark, Germany, Spain) testing a smartphone self-management app for depression. Participants are randomized to active rule-based feedback vs. monitoring-only control. The rule-based feedback is generated based on self-monitored data and sensor data collected from the smartphones. The purpose of this rule-based feedback is to suggest supportive actions to participants, such as reading items from a content library within the app or contacting emergency healthcare facilities (75, 76).

### 3.1 SWOT analysis

**Figure 3:**
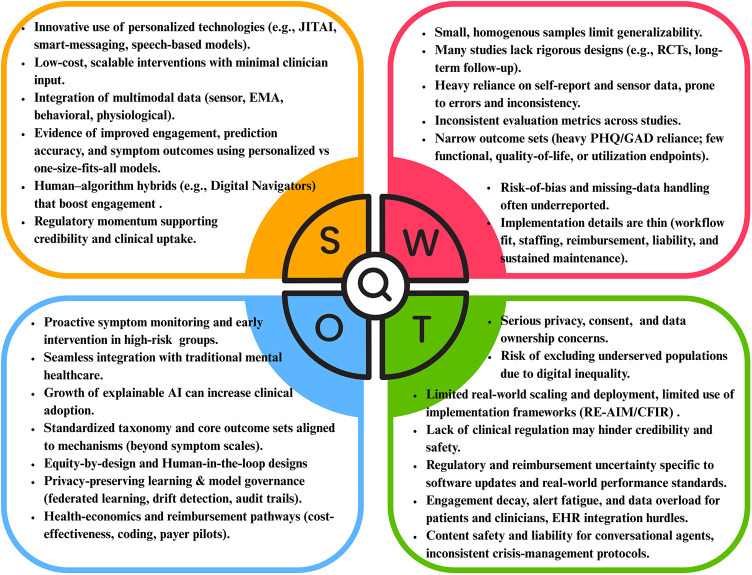
SWOT analysis of reviewed literature.

Personalized digital interventions for depression show strong potential due to their scalability, multimodal integration (e.g., EMA, sensors), and promising engagement outcomes. Opportunities include early intervention, Explainable Artificial Intelligence (XAI), and integration with traditional care. However, weaknesses such as small samples, inconsistent evaluation, and reliance on self-report data persist. Major threats include data privacy issues and digital exclusion of underserved populations. The SWOT results are shown in Figure 3.

### 3.2 Risk of bias assessment

**Figure 4:**
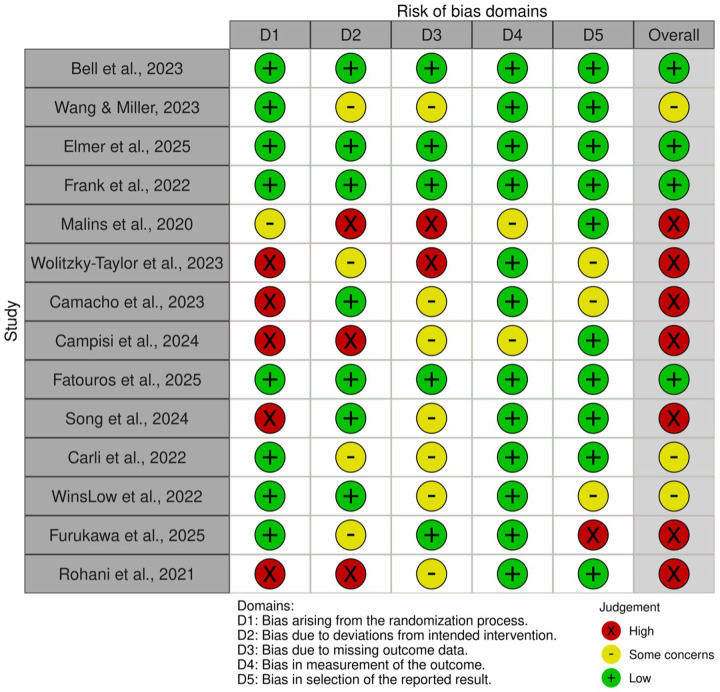
Rob 2.0 assessment of prospective studies.

4 studies (19, 51, 53, 60) showed a low overall risk of bias, as shown in Figure 4. Most others raised concerns, especially regarding randomization, missing data, and selective reporting. The predictive models assessed in the 3 retrospective studies were found to exhibit low to intermediate risk of bias, as shown in Table 5. These limitations highlight the need for more rigorous designs and standardized outcome reporting in future digital mental health research.

**Table 5:** PROBAST results.

Study	D1 Participants		D2 Predictors			D3 Outcome						Overall
	1.1	1.2	2.1	2.2	2.3	3.1	3.2	3.3	3.4	3.5	3.6	
Gerczuk et al. (17)	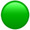	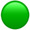	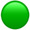	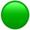	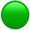	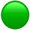	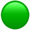	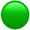	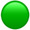	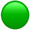	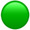	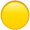
Chatterjee et al. (49)	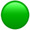	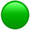	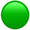	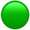	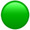	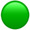	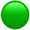	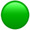	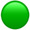	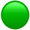	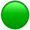	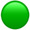
Kathan et al. (48)	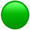	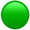	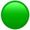	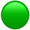	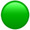	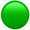	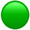	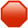	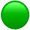	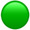	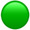	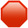
Study		D4 Analysis									Overall	
		4.1	4.2	4.3	4.4	4.5	4.6	4.7	4.8	4.9		
Gerczuk et al. (17)		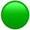	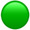	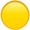	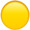	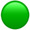	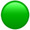	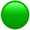	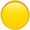	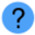	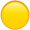	
Chatterjee et al. (49)		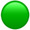	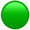	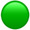	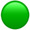	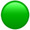	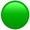	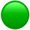	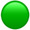	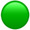	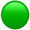	
Kathan et al. (48)		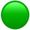	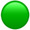	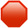	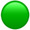	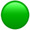	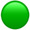	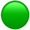	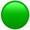	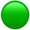	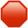	
D1: 1.1 Appropriate data sources used?; 1.2 Inclusions/exclusions appropriate? D2: 2.1 Predictors defined & assessed uniformly?; 2.2 Predictor assessment blinded to outcome?; 2.3 Predictors available at intended use? D3: 3.1 Outcome determined appropriately?; 3.2 Pre-specified/standard outcome definition?; 3.3 Predictors excluded from outcome definition?; 3.4 Outcome measured uniformly?; 3.5 Outcome assessment blinded to predictors?; 3.6 Time interval appropriate? D4: 4.1 Reasonable number of participants with outcome?; 4.2 Continuous/categorical predictors handled appropriately?; 4.3 All enrolled participants included in analysis?; 4.4 Missing data handled appropriately?; 4.5 Univariable predictor screening avoided?; 4.6 Complexities (e.g., censoring, competing risks) appropriately handled?; 4.7 Relevant performance measures evaluated appropriately?; 4.8 Overfitting & optimism accounted for?; 4.9 Predictor weights correspond to reported analysis?										Judgement 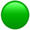 Yes 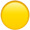 Probably not 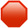 No 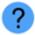 No information		

Across three non-scored narrative appraisals, Woebot (WB001), MindBot, and HAYT are clearly described but differ in clinical maturity and evidentiary strength. WB001 provides the most complete clinical framing, an FDA Breakthrough, prescription 8-week Agent-delivered CBT and interpersonal therapy program with daily mood tracking and NLP-based crisis detection, yet the brief lacks detailed data-governance disclosures (13). MindBot offers a rigorous engineering overview (preprocessing, sentiment thresholds, LLM-augmented replies), but it provides limited bias-monitoring and escalation specifics (46). HAYT delineates a clinician-integrated workflow (NLP of diary entries, DSM-5 questionnaires, CBT prompts, secure messaging) with transparent data flow in principle, though results are based on synthetic data and do not validate real-world safety or clinical impact (47). Across all three, safety provisions are conceptually present but unspecified in performance terms. Credibility would be strengthened by explicit reporting on privacy, human-in-the-loop escalation, and algorithm update policies.

In terms of transparent research practices and open scientific publication, we observed heterogeneous adoption of open-science practices across the corpus. Trial registration was common in RCTs, but public protocols, pre-specified analysis plans, de-identified data, and analysis code were infrequently shared in most studies. We therefore flag transparent preregistration (e.g., ClinicalTrials.gov/OSF), protocol publication, and routine data- and code-sharing with privacy safeguards as concrete steps to reduce selective-reporting risk and improve reproducibility in this rapidly evolving field.

## 4 Discussion

Depression represents a multifaceted neuropsychiatric condition characterized by pronounced phenotypic heterogeneity across symptomatology, etiology, pathophysiology, and treatment response trajectories. This intrinsic variability manifests through diverse clinical presentations, ranging from predominantly somatic manifestations to primarily cognitive dysfunctions, thereby challenging the efficacy of standardized therapeutic approaches. Contemporary nosological frameworks such as the DSM-5 provide categorical diagnostic parameters; however, these fail to capture the dimensional complexity of depressive phenomenology as revealed through advanced digital phenotyping methodologies and precision psychiatry initiatives.

The suboptimal efficacy of conventional interventions may be attributed to their inability to accommodate inter-individual variability in symptom constellations, neurobiological substrates, and psychosocial determinants. This problematic homogenization of heterogeneous depressive states necessitates a paradigmatic shift toward personalized intervention strategies informed by multimodal assessment protocols and computational modeling techniques.

**Figure 5:**
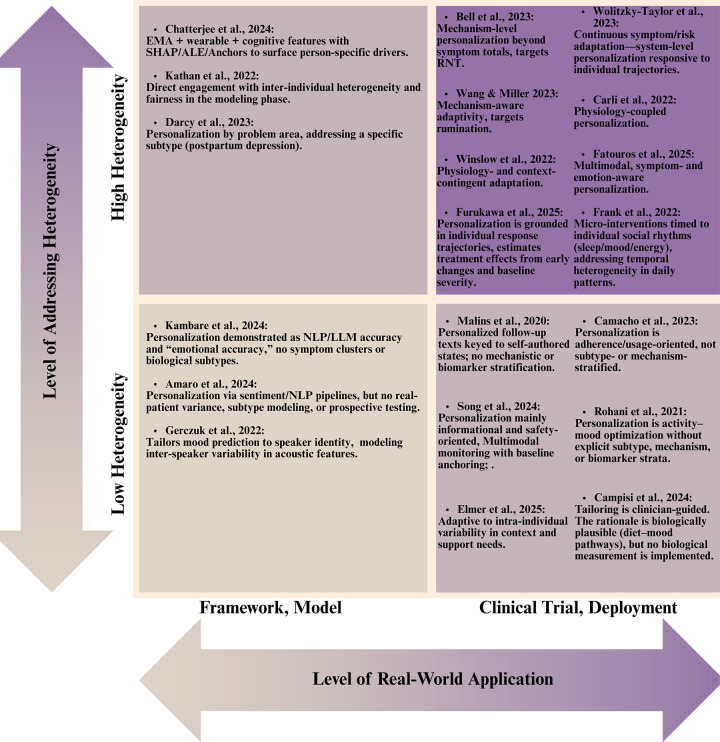
How heterogeneity is addressed in the literature.

The examined literature corpus demonstrates variable engagement with depression heterogeneity, reflecting a spectrum of methodological sophistication in addressing inter-individual and intra-individual symptom variability. Here, a two-dimensional evaluation framework (Figure 5) is proposed to assess the studies according to: (1) their level of heterogeneity engagement (low vs. high) and (2) their implementation stage (model building vs. clinical deployment). This taxonomic approach reveals significant disparities in how depression heterogeneity is operationalized across the research spectrum.

### 4.1 Models with limited heterogeneity integration

Gerczuk et al. (17) personalize mood estimation to speaker identity, capturing inter-speaker variance in acoustic features, yet remain uncustomized to symptom mechanisms (17). MindBot and HAYT demonstrate NLP and LLM-assisted pipelines and, in HAYT's case, a clinician-messaging architecture using synthetic data (46, 47). These works illustrate that personalization can be embedded at the interface layer; nonetheless, they largely bypass clinical variance, no discussion of clinical endpoints, no subtype stratification, and limited attention to safety, governance, or model drift. Proxy metrics (e.g., “emotional accuracy”) and synthetic diaries risk overstating clinical readiness while under-representing failure modes in non-standard language, high-distress states, or low-literacy populations.

### 4.2 Clinical applications with limited heterogeneity integration

These studies personalize primarily by schedule, adherence support, or single-channel signals. Rohani et al. (56) evaluated recommendation algorithms for pleasant event scheduling across clinical and non-clinical populations. While their approach accommodates intra-individual variability in activity-mood associations, it does not explicitly model symptom heterogeneity or depression subtypes, instead focusing on behavioral intervention optimization through reinforcement learning principles (56).

Malins et al. (54) investigated personalized relapse prevention messaging CBT, with messages tailored to individual recovery trajectories across distinct clinical states (wellness maintenance, early warning signs, full relapse). This strategy acknowledges heterogeneity in relapse vulnerability and symptom recognition patterns, though it relies on clinician-guided personalization without mechanistic or biomarker stratification (54). The m-Path social-support JITAI tailors timing and content via self-signaled need and momentary affective context (19). Adaptation is person-specific but not mechanism- or biotype-aware. This creates person-level adaptation on two axes: detection thresholds based to individual baselines and action menus refined by user preference. Yet the mechanism model remains indifferent to subtype. There is no personalization by symptoms, no biophysical state inputs, and no contextual signals (19).

Personalization in the living-lab for socially vulnerable older adults is primarily achieved through individualized baselining and longitudinal feedback rather than just-in-time control (50). Participants wear multimodal sensors (e.g., heart-rate variability, sleep efficiency/fragmentation, activity) and complete high-frequency chatbot PHQ check-ins. These streams are harmonized to compute each person's baseline and day-over-day deltas for mood and physiology. Personalization therefore resides in the personal reference model and tailored visualizations that support self-care and caregiver awareness. However, there is no policy that optimizes timing or content based on estimated treatment effects, and no stratification by symptom dimension or biotype (50).

Digital-navigator coaching personalizes engagement rather than content; coaches review mindLAMP activity and symptom self-reports and then tailor next steps like what module to try next and which homework to emphasize, via human-in-the-loop heuristics (55). Adaptation is driven by recent completion patterns, working alliance scores, and user-stated goals, with cadence modulated to sustain adherence. This raises dose and breadth of app use at the person level but personalization is pragmatic, not mechanistic (55).

The personalized nutrition program for adolescent MDD individualizes targets at three layers: diet goals, eating-behavior, and a weekly menu plan tuned to family preferences and shopping logistics (61). Although the intervention is biologically plausible, leveraging Mediterranean-style patterns to influence inflammation, gut–brain signaling, and metabolic rhythms, the personalization engine does not measure or stratify by biological markers like CRP or microbiota, circadian rythm, or cognitive symptoms. As a result, “biology-aware” matching of diet to patient is not implemented and personalization remains preference-based rather than biology-directed (61).

Most of the reviewed clinical trials implement personalization strategies centered primarily on behavioral parameters (e.g., mood fluctuations, patient-reported outcomes, application engagement metrics) or symptom severity indices, without establishing explicit linkages to biological subtypes or neurobiological mechanisms. CRM forecasts personalized next-day mood from wearables and check-ins to trigger risk-contingent prompts (NCT05400785) (63). The response-based transdiagnostic internet trial adapts treatment format and dose by using early symptom trajectories to add brief therapist sessions for late responders (NCT07051148) (65). mHELP combines ML stress detection on phone and smartwatch streams with on-demand skills and two light telehealth touchpoints (NCT07017569) (64). MENTINA applies personalized rule-based feedback from questionnaire and sensor data, escalating to safety guidance when indicated (NCT06919133) (75). These approaches, while clinically valuable, conceptualize depression predominantly as a psychological or behavioral syndrome, neglecting to differentiate or specifically target distinct biological subtypes that may respond differentially to intervention strategies.

### 4.3 Models with advanced heterogeneity integration

Chatterjee et al. (49) implemented explainable deep learning architectures for mood prediction using multimodal data streams (EMA, physiological parameters, cognitive assessments). Their methodological innovation lies in the application of interpretability techniques (SHAP, ALE, Anchors) to elucidate person-specific predictors of mood disturbance. This approach enables computational phenotyping aligned with natural symptom expression, though it does not explicitly classify clinical subtypes or stratify based on symptom dimensions (49). On the other hand, the Woebot WB001 device profile engages a specific clinical subtype (postpartum depression), illustrating how tailoring by life context can anchor personalization even at the caregiving stage (13).

Kathan et al. (48) conducted a comparative evaluation of multiple personalization strategies for symptom prediction, including transfer learning with shared and individualized components, subject-specific data preprocessing, and gender-based stratification. Their explicit assessment of model equity across demographic subgroups represents one of the few studies directly addressing fairness in computational personalization. This is a critical consideration given the documented disparities in depression presentation across demographic strata. Their multifaceted approach to heterogeneity encompasses both methodological innovation and equity considerations (48).

Together, these studies show that individualized models outperform pooled baselines and that equity assessment is feasible, marking a substantive step beyond undifferentiated prediction. However, they stop short of prospective decision rules: neither specifies thresholds that trigger action, nor do they test whether model outputs change behavior, adherence, or outcomes in real life. Small, intensively monitored cohorts also risk selection bias and limit generalizability. Moreover, depressive mechanisms are largely statistical rather than biological, in other words, predictive features are not validated against circuitry, inflammatory markers, or subtype taxonomies.

### 4.4 Clinical applications with advanced heterogeneity integration

Several interventions tailor when and what to deliver using mechanisms that vary across people. Bell et al. (51) evaluated the Mello application, which delivers personalized cognitive-behavioral interventions based on real-time assessment of mood, rumination, location, and activity. By targeting a transdiagnostic mechanism, RNT, that presents with substantial inter-individual variability, their approach shows sophisticated engagement with heterogeneity beyond symptomatic expression. The intervention's significant efficacy (*d* = 0.50 for depression, *d* = 0.61 for anxiety, *d* = 0.87 for RNT) and mediation findings support the clinical utility of mechanism-focused personalization strategies (51). Similarly, the JITAI by Wang & Miller (52) measures receptivity and targets rumination, showing medium-to-large effects and demonstrating that targeting heterogeneous cognitive processes can outperform symptom-total heuristics (52). Still, mechanisms are inferred from self-reports, passive indicators that could address cognitive states (speech, mobility, physiology) would add more mechanistic information.

Across other programs, personalization is implemented through distinct sensing and decision layers. Cue derives an individualized “social rhythm” baseline from passive smartphone traces (sleep–wake cycle, mobility, communication patterns) augmented by brief mood/energy check-ins. Deviations from that baseline trigger rule-mapped micro-interventions at a cadence designed to minimize alert fatigue by suppressing and rotating content and timing based on recent engagement. While this addresses symptom level and temporal heterogeneity, a fuller account should discuss biological correlates, particularly circadian regulation, as mechanistic levers for personalization (53).

Physiology-coupled systems (NEVERMIND, FeelDTx, smartwatch-CBT) learn per-user baselines of HR and HRV, electrodermal activity, sleep efficiency and fragmentation, temperature and activity; state detectors (rule-based or lightweight ML) fire context-appropriate prompts (paced breathing/HRV biofeedback for sympathetic arousal, mindfulness or reframing during negative-affect windows, sleep-hygiene guidance nocturnally) with refractory periods and weekly navigator/therapist touchpoints where applicable (58–60). While the addition of physiological markers introduces symptomatic and temporal heterogeneity to the intervetion, future iterations should incorporate biotype-aware targeting, subgroup calibration, and fairness audits to raise the overall level.

STAND and RESiLIENT operationalize personalization at the system level, addressing heterogeneity in treatment effects even if not biomarker-defined. Through repeated symptom and suicide-risk assessments, the STAND algorithm assigns the user to one of stepped tiers of care with real-time alerts and scripted escalation pathways (57). The RESiLIENT models use baseline PHQ-9 patterns and early symptoms to estimate individual treatment effects, then assign the “best next” skill block (15).

Multiple trials showed sophisticated engagement with neurobiological heterogeneity, representing a paradigmatic advancement in precision psychiatry. The Digital Therapeutics for Apathy in Late-life Depression trial (NCT05877885) (72) specifically targets network dysfunction underlying apathy, a clinically distinct dimension frequently observed in geriatric depression presentations. The intervention architecture is meticulously designed to modulate specific neural circuitry, particularly networks subserving attention allocation, salience detection, and cognitive control functions; all systems that are consistently implicated in motivational deficits across neuroimaging and neurobiological investigations.

Similarly, the FDA-regulated clinical trial Effectiveness of Digital Therapeutics in Major Depressive Disorder (74) evaluates *Rejoyn*, a digital therapeutic explicitly engineered to engage neural networks implicated in major depressive psychopathology, with particular emphasis on neuroplasticity mechanisms. Unlike conventional approaches that conceptualize depression as a unitary construct, this trial acknowledges fundamental neural circuitry heterogeneity in MDD, implementing structured neurobehavioral exercises specifically designed to recalibrate dysfunctional neural circuits. Notably, the trial documentation explicitly addresses limitations of traditional pharmacological interventions that primarily target neurochemical dysregulation, highlighting the critical importance of circuit-based therapeutic approaches.

The MASE project personalizes timing and content by learning each person's Activity–Subjective Energy Association from accelerometry and ecological diaries, then combining this with neurobiological profiling to prescribe micro-activities matched to an individual's brain phenotype (NCT07059234) (62). REJUVENATE adapts task parameters and progression rules across multiple cognitive domains using in-app telemetry, aiming to remediate person-specific cognitive control deficits via neuroplasticity-based training (NCT04961047) (66). MEL-T01 embeds continuous performance sensing into gameplay to dynamically adjust difficulty, stimuli, and reinforcement schedules to an individual's executive-function profile (NCT05426265) (70).

Collectively, these studies operationalize heterogeneity at multiple levels, behavioral dynamics, cognitive phenotype, and neural circuitry, thus moving beyond severity-only tailoring. Methodological challenges remain (e.g., ensuring reliability of neurobehavioral markers, external validity across biotypes, and fairness audits for model-guided decisions), but the personalization mechanisms are technically rich and mechanism-aligned.

### 4.5 Methodological limitations and implementation challenges

Despite promising advances, several critical limitations persist across the reviewed literature:

- Limited Integration of Established Subtypes

Few studies explicitly incorporate validated depression subtypes (e.g., melancholic, atypical, anxious) or empirically derived symptom dimensions into their personalization frameworks. This disconnect between clinical phenotyping research and digital intervention development represents a significant translational gap that restricts mechanistic specificity and may obscure heterogeneous treatment effects across clinically meaningful subgroups.

- Predominance of Unidimensional Outcome Measures

Many studies rely on composite mood scores or generalized depression severity metrics (e.g., PHQ-9, PHQ-8) rather than multidimensional symptom assessments. This approach potentially obscures differential effects on specific symptom clusters, such as sleep dysregulation, anergia, or cognitive slowing, that may vary across depression subtypes. Symptom-network analyses and domain-specific endpoints would better capture mechanism-aligned change and enable more nuanced estimation of heterogeneous treatment effects.

- Sample Limitations and Generalizability of Machine-Learning Findings

Across studies, samples are typically small (often *N* < 100), convenience-based, and demographically narrow, which amplifies overfitting risk and limits transportability of findings. Reported performance gains typically reflect within-sample or cross-validated performance against naïve baselines rather than out-of-distribution performance across settings, devices, or clinical strata.

- Insufficient Attention to Algorithmic Equity

With the notable exception of Kathan et al. (48), most studies inadequately address potential algorithmic biases across demographic and clinical subgroups. Few models report disaggregated performance metrics by gender, age, race/ethnicity, or socioeconomic status, nor do they examine differential false-alert rates or error patterns that could exacerbate existing health disparities. Given well-documented differences in depression presentation, help-seeking behavior, and digital access across demographic contexts, this represents a critical oversight. Routine fairness diagnostics, including performance parity, error symmetry, and calibration equity analyses, should be standard practice, accompanied by corrective strategies (e.g., stratified sampling, group-aware decision thresholds, re-weighting) and transparent reporting of any performance-fairness trade-offs.

- Limited External Validation and Deployment Science

Even prototype systems demonstrating efficacy within controlled research settings lack replication across independent health systems, diverse payer contexts, and real-world clinical workflows. Implementation outcomes such as reach, adoption fidelity, maintenance, and cost-effectiveness are consistently underreported. Without integration of established implementation science frameworks (for example, the Reach, Efficacy, Adoption, Implementation, and Maintenance framework (RE-AIM) (22) and the Consolidated Framework for Implementation Research (CFIR) (18)) and inclusion of payer-relevant endpoints, questions of scalability and sustainability remain unanswered. Prospective evaluations should ideally embed models within actual clinical workflows through silent deployment or randomized alert configurations to estimate real-world effectiveness and detect performance drift over time.

- Inadequate Temporal Resolution

Despite theoretical emphasis on dynamic symptom fluctuation, many studies employ relatively sparse assessment protocols that fail to capture rapid symptom oscillations potentially indicative of specific depression subtypes or vulnerability patterns. Current sampling cadences often miss context switches, diurnal mood variations, and event-triggered state changes that are critical for just-in-time adaptive interventions. Future designs should favor denser, temporally aligned multimodal data collection with principled handling of missingness through pattern diagnostics and multiple imputation methods tailored to time-series contexts.

### 4.6 Future directions and methodological recommendations

Future work must begin by grounding personalization algorithms in empirically derived depressive subtypes. Rather than treating depression as a unidimensional construct, researchers could apply unsupervised learning techniques to multimodal datasets (77). These data-driven subgroups can then inform model architectures that tailor predictions to the unique symptom constellations of melancholic, atypical, anxious, or other clinically meaningful clusters. At the same time, interventions should move beyond composite mood scores and incorporate multidimensional symptom assessments, using network-analysis approaches to map the dynamic interrelationships among symptoms (78). By capturing how fatigue, anhedonia, sleep disturbance, and cognitive dysfunction co-activate and cascade over time, digital tools can offer more precise, subtype-specific feedback and treatment recommendations. Following the promising approaches discussed earlier, interventions should prioritize targeting transdiagnostic mechanisms with established heterogeneity in addition to general behavioral patterns and preferences.

Progress will hinge on moving beyond small, convenience samples toward adequately powered, diverse, and prospectively enrolled cohorts. Multi-site recruitment with stratified targets (age, sex/gender, race/ethnicity, socioeconomic status, language, device/OS) should be pre-specified and monitored. To curb overfitting and analytic bias, modeling work should adopt participant-level, temporally blocked, nested cross-validation and report both discrimination (e.g., MAE/RMSE, AUROC) and calibration (slope, intercept, expected calibration error).

Equity must become a core design principle rather than an afterthought. Future personalization frameworks should adopt fairness-aware optimization strategies—such as demographic parity or equalized odds constraints—to ensure consistent performance across gender, age, and cultural groups (79). Algorithmic bias can systematically skew who benefits and who is harmed. Disparate error rates (e.g., higher false positives for crisis alerts in one group, higher false negatives in another) distort triage, amplify clinician workload unevenly, and may delay care for those already underserved. Interventions would benefit from routine bias audits that quantify disparities in predictive accuracy and treatment suggestions, followed by algorithmic recalibration where needed (80). In parallel, there is a need for consensus on reporting standards, research protocols ought to include a “heterogeneity specification” checklist that details how subtypes were defined, which symptom dimensions were assessed, and what bias-mitigation techniques were implemented. Such standardized reporting will facilitate cross-study comparison, meta-analysis, and the cumulative advancement of the field. Moreover, to strengthen transparency and reproducibility, depression-focused digital trials should adopt open-science practices such as preregistration and open sharing of code and de-identified data. Practical steps like registering hypotheses and analysis plans before data collection, archiving code and data in trusted repositories, and publishing preprints, are feasible now and would materially improve credibility in this fast-moving field (81, 82).

Additionally, truly user-centered innovation requires embedding human-centered design (HCD) throughout the development lifecycle. Researchers should engage patients, clinicians, and caregivers in participatory co-design workshops, iteratively refining wireframes, feature sets, and interaction flows based on real-world feedback (83, 84). Usability testing, employing think-aloud protocols and standardized measures (e.g., the System Usability Scale), can uncover interaction bottlenecks before large-scale deployment. Accessibility and inclusivity audits, evaluating readability, language support, digital literacy, and disability accommodations, will further ensure that personalized digital mental health tools are equitable and resonate with diverse user populations. By integrating robust analytics, fairness safeguards, and rigorous HCD practices, the next generation of interventions can fulfill the promise of precision psychiatry in a way that is both scientifically sound and deeply humane.

Finally, clinical integration should move beyond app availability to workflow-embedded, measurement-based care. Health systems can operationalize digital tools through (i) EHR-integrated screening and triage that route patients to matched interventions; (ii) a defined digital navigation role for onboarding, troubleshooting, and engagement support; and (iii) scheduled reassessment checkpoints that trigger escalation, switching, or augmentation within stepped-care pathways (85, 86). Interoperability and clear reimbursement pathways are critical to sustain routine use. Implementation should be guided by RE-AIM/CFIR with pragmatic and adaptive trials embedded in care to learn which components drive benefit (87). Systems must add equity and safety guardrails, like stratified performance rates, multilingual/low-literacy designs, transparent data-governance, and ethical frameworks. Finally, routine workload and cost accounting should inform scalable resourcing decisions (85, 86). Together, these steps shift digital interventions from promising pilots to reliable, equitable infrastructure for depression care.

## 5 Conclusion

The heterogeneous nature of depression necessitates personalized intervention approaches that accommodate inter-individual variability in symptom presentation, etiological factors, and treatment response patterns. Digital mental health technologies offer unprecedented opportunities for implementing such precision approaches at scale, yet current methodologies demonstrate variable engagement with heterogeneity, from superficial customization to sophisticated computational phenotyping.

The most promising approaches, exemplified by Bell et al. (51), Wang and Miller (52), and Frank et al. (53), integrate advanced computational methods with clinically informed conceptualizations of depression heterogeneity. By targeting transdiagnostic mechanisms, employing interpretable ML, and evaluating algorithmic equity, these studies point toward a future where digital interventions can truly accommodate the multidimensional nature of depressive psychopathology.

However, significant methodological challenges remain, particularly regarding the integration of established clinical phenotypes, multidimensional outcome assessment, and cross-population validation. Addressing these limitations will require interdisciplinary collaboration between clinical researchers, computational scientists, and implementation specialists to ensure that technological innovations translate into meaningful clinical outcomes across the heterogeneous spectrum of depressive disorders.

## Data Availability

The original contributions presented in the study are included in the article/Supplementary Material, further inquiries can be directed to the corresponding author/s.

## Glossary

ALE: accumulated local effects
BDNF: brain-derived neurotrophic factor
BMI: body mass index
CBT: cognitive behavioral therapy
CFIR: consolidated framework for implementation research
COVID-19: coronavirus disease
CRH: corticotropin-releasing hormone
CRP: C-reactive protein
DALYs: disability-adjusted life years
DL: deep learning
DMN: default mode network
DSM-5: diagnostic and statistical manual of mental disorders, fifth edition
EDA: electrodermal activity
EEG: electroencephalography
EHR: electronic health record
EMA: ecological momentary assessment
FDA: U.S. Food and Drug Administration
GABA: gamma-aminobutyric acid
GAD-7: generalized anxiety disorder-7
HCD: human-centered design
HPA: hypothalamic–pituitary–adrenal axis
HR: heart rate
HRV: heart rate variability
IL-6: interleukin-6
IL-8: interleukin-8
JITAI: just-in-time adaptive intervention
KYNA: kynurenic acid
LC: locus coeruleus
LLM: large language model
MAO: monoamine oxidase
MASE: motor activity–subjective energy
MBC: measurement-based care
MDD: major depressive disorder
ML: machine learning
NE: norepinephrine
NICE: National Institute for Health and Care Excellence
NLP: natural language processing
PHQ-9: patient health questionnaire-9
PRISMA: preferred reporting items for systematic reviews and meta-analyses
PROBAST: prediction model risk of bias assessment tool
PRS: polygenic risk score
QUIN: quinolinic acid
RCT: randomized controlled trial
RE-AIM: reach, efficacy, adoption, implementation, and maintenance
RNT: repetitive negative thinking
RoB: risk-of-bias tool for randomized trials
SHAP: SHapley Additive exPlanations
SMS: short message service
SNP: single nucleotide polymorphisms
SVM: support vector machine
SWOT: strengths, weaknesses, opportunities, threats
TNF-α: tumor necrosis factor alpha
TRD: treatment-resistant depression
WBC: white blood cells
XAI: explainable artificial intelligence.
